# Determining the susceptibility of carbapenem resistant *Klebsiella pneumoniae* and *Escherichia coli* strains against common disinfectants at a tertiary hospital in China

**DOI:** 10.1186/s12879-020-4813-6

**Published:** 2020-01-30

**Authors:** Yili Chen, Kang Liao, Yongxin Huang, Penghao Guo, Han Huang, Zhongwen Wu, Min Liu

**Affiliations:** 1 Department of Laboratory Medicine, The First Affiliated Hospital of Sun Yat-sen University, Guangzhou, 510080 Guangdong China; 2 Guangdong Province Key Laboratory of Malignant Tumor Epigenetics and Gene Regulation, Research Center of Medicine, Sun Yat-Sen Memorial Hospital, Sun Yat-Sen University, Guangzhou, 510080 Guangdong China

**Keywords:** Carbapenem resistant, *Klebsiella pneumonia*, *Escherichia coli*, Disinfectant, susceptibility

## Abstract

**Background:**

Carbapenem-resistant *Enterobacteriaceae* (CRE) infections have become a global health threat. Controlling CRE transmission in hospitals is increasingly dependent on the use of disinfectants to restrict the risk of infection. Here, the susceptibility of patient-derived carbapenem resistant *Klebsiella pneumoniae* (CRKP) and *Escherichia coli* (CREC) strains against three common disinfectants and the determinants of resistance to disinfectants were investigated.

**Methods:**

The minimum inhibitory concentrations (MICs) and the minimum bactericidal concentrations (MBCs) of three common chemical disinfectants: chlorhexidine, trichloroisocyanuric (TCCA) acid and Povidone iodine (PVP-I) against 50 CRE strains were measured. The drug-resistance genes -*qacEΔ1*, *qacA/B* and *cepA*-were determined using polymerase chain reaction.

**Results:**

A total of 36 CRKP and 14 CREC strains were collected in our hospital from 2016 to 2018. The MIC ranges of 36 CRKP strains against chlorhexidine, TCCA and PVP-I were 8~512 mg/L, 64~128 mg/L and 8~128 mg/L, respectively. For 14 CREC strains, the MIC ranges against chlorhexidine, TCCA and PVP-I were 4~128 mg/L, 64~128 mg/L and 4~128 mg/L, respectively. Moreover, against chlorhexidine and PVP-I, the MIC_90_ of 36 CRKP strains was higher than that of 50 CSKP strains. The *qacE△1* gene was detected in 15 isolates among 36 CRKP strains (41.7%), and 8 isolates among 14 CREC strains (57.1%); while the *qacA/B* gene was not detected. Specifically, the *cepA* gene was much more prevalent than the *qacEΔ1*; it reached over 80% among CRKP strains. Compared to the CSKP strains, the presence of the *qacEΔ1* and *cepA* genes was significantly higher among the CRKP strains (*p < 0.05*).

**Conclusion:**

CRE strains collected from patients in our hospital exhibit various degree of resistance to the commonly used chemical disinfectants. It is of great help to keep monitoring the tendency of the reduced susceptibility of the pan-resistant strains against disinfectants, in order to effectively control and prevent the spread of the super resistant bacteria.

## Background

Carbapenem-resistant *Enterobacteriaceae* (CRE) is a globally important nosocomial pathogen. Infections caused by CRE are associated with increased morbidity and mortality rates and greater hospital costs [1, 2]. CRE-infected patients often suffer multiple underlying diseases and are in immunosuppression. Effective drugs to eliminate this infection from the patients are limited currently. Therefore, strategies to prevent initial infection by eliminating or at least reducing the presence of this bacteria in the clinical environment is of significant importance, and should be given a high priority by clinicians [3].

Disinfectants are extensively applied to control infectious organisms from potentially contaminated equipment and specimens. However, like the emergence of antibiotic resistance, drug-resistant bacteria may gradually become resistant to the commonly used clinical chemical disinfectants, especially because of the possible similar mechanisms between antibiotic resistance and disinfectant resistance [4]. The excessive use of disinfectants imposed selective pressure on strains, causing a wide distribution of disinfectant resistance genes. Many disinfectant resistance genes have been confirmed in multidrug-resistant bacteria, such as *qacA/B, qacE, qacEΔ1, qacG, qacJ, cepA, arcA and kdeA* [5–7].

Effectiveness of disinfectants against *Enterobacteriaceae* was reported previously [8–10]. However, less information about disinfectant effectiveness against carbapenem-resistant *Enterobacteriaceae* (CRE) is available. In this study, carbapenem resistant *Klebsiella pneumoniae* (CRKP) and *Escherichia coli* (CREC) strains were isolated from patients at the First Affiliated Hospital of Sun Yat-sen University. The minimum inhibitory concentration (MIC) and minimum bactericidal concentration (MBC) of the commonly used disinfectants against each CRE strain were determined. Moreover, the presence of relevant resistance genes was determined.

## Methods

### Isolation and identification of bacterial strains

A total of 36 CRKP and 14 CREC strains with ertapenem MICs≥2 μg/ml were collected in the First Affiliated Hospital of Sun Yat-sen University from 2016 to 2018. Meanwhile, 50 strains of carbapenem susceptible *Klebsiella pneumoniae* (CSKP) and 30 strains of carbapenem susceptible *Escherichia coli* (CSEC) were collected as control group strains (ertapenem MICs≤0.5 μg/ml). Clinical specimens were collected from urine, blood, sputum, sterile body fluid and wound secretion. Identification of isolates was performed using an automated microbiology analyzer (bioMérieux, Marcy l’Etoile, France) according to the manufacturer’s instructions. The standard strains included *Klebsiella pneumoniae* ATCC 700603 and *Escherichia coli* ATCC 25922.

### Antimicrobial susceptibility testing

Antimicrobial susceptibilities for isolates were detected initially by Gram-negative susceptibility (GNS) cards on the Vitek system (bioMérieux, Marcy l’Etoile, France). Antimicrobials evaluated included piperacillin-tazobactam, ampicillin-sulbactam, levofloxacin, ceftriaxone, ceftazidime, cefotaxime, imipenem, cefepime, ampicillin /clavulanic acid cefoxitin, ciprofloxacin, and amikacin. Susceptibility testing results were interpreted under the criteria recommended by the Clinical and Laboratory Standards Institute (CLSI, 2018). The quality control strain for susceptibility testing was *E. coli* ATCC 25922.

### PFGE

Pulsed-field gel electrophoresis (PFGE) analysis was performed as described previously with the *XbaI* restriction endonuclease (TAKARA, Shiga, Japan) [11] and the Fingerprinting II Informatix software package system (Bio-Rad Laboratories, Hercules, CA). The similarity of the PFGE banding patterns was calculated by the Dice coefficient, and the data acquired were carried out by the unweighted pair group method with arithmetic average (UPGMA) clustering by the Pearson correlation coefficient.

### Disinfectants and neutralizers

In the study, three disinfectants were used, and they were 0.1% chlorhexidine (Chinese Co., Ltd., Jinzhou, China), trichloroisocyanuric acid (TCCA) (Changjiang Mai Medicine Technology Co., Ltd., Beijing, China) and 0.1% Povidone iodine (PVP-I) (An Duo Fu, Shenzhen, China). Table 1 shows the neutralizing agents used to inhibit each of the disinfectants.

**Table 1:** Neutralizers used to neutralize the three chemical disinfectants respectively

Disinfectants	Neutralizers
0.1% chlorhexidine	5.0% Tween 80
TCCA	1000 ml PBS + 5 g sodium thiosulfate + 0.5% Tween 80
0.1% PVP-I	1000 ml PBS + 10 g sodium thiosulfate + 1.0% Tween 80

### Testing the MICs and MBCs for the effectiveness of each disinfectant

MIC_S_ (minimum inhibitory concentrations) of the three disinfectants against CRKP and CREC clinical isolates were determined by micro-broth dilution method according to the guidelines of the CLSI (CLSI, 2018), in concentrations that ranged from 1~512 mg/L for chlorhexidine, 2~1024 mg/L for TCCA, and 1~512 mg/L for 0.1% PVP-I. Firstly, the standard bacterial concentration of McFarland standard 0.5 was applied (1.5✖10^8^ cfu/mL). The 0.5 McFarland inoculum suspensions were further diluted at 1: 100 in Luria Broth (LB) before inoculation. 50 μL of bacterial suspension was added from wells 1 to 12 in a 96-well plate, followed with 50 μlL chlorhexidine, TCCA or 0.1% PVP-I. LB without disinfectant was inoculated with the bacteria and used as the positive control, while LB alone was used as the negative control. The plates were incubated at 37 °C overnight. After the 24 h incubation for MIC determination, the reactions from the above MIC tests that did not exhibit bacterial growth were selected, and 0.05 mL of the sterile reaction was transferred into 0.45 mL neutralizer specific for the particular disinfectant used in each test. The solution was mixed thoroughly and incubated at room temperature for 10 min as the final reaction solution. 0.5 mL of the each mixed solution was used to coat in a MH agar plate. Samples were incubated at 37 °C for 24 h. The minimum concentration of the disinfectant corresponding to the sterile plate was determined to be the MBCs (minimum bactericidal concentrations) of the disinfectant against the tested bacterial strain. The positive and negative control groups were prepared as described above in the MIC experiment, and solutions containing 0.45 mL of a neutralizer plus 0.05 mL of the LB were used as the controls for the neutralizers. Experiments were performed in triplicate, with consistent results.

### PCR detection and sequence analysis of resistance genes

Bacterial DNA was extracted from CRKP and CREC isolates by boiling. PCR of resistance genes *qacEΔ1*, *qacA/B* and *cepA* was performed using TaKaRa Ex Taq (Takara Bio Inc., Otsu, Japan) on the Applied Biosystems® 7500 Fast Dx Real-Time PCR Instrument (Life Technologies Corporation, Foster City, CA). All PCR primers targeting resistance genes used in this study are listed in Table 2. Appropriate positive and negative controls for amplification were selected from clinical *Klebsiella pneumoniae* isolates. The positive controls that carried the resistance genes were confirmed using PCR followed by sequence analysis. Each 20 μL PCR tube included 2 μL DNA template, 6 μL sterile water, 1 μL forward primer (Sangon Biotech), 1 μL reverse primer and 10 μL 2✖ Taq Master Mix (Takara Bio Inc., Otsu, Japan). The PCR conditions was set as follows: 94 °C for 5 min, followed by 30 cycles of 94 °C for 30 s for denaturation, 53 °C for 30 s for annealing and 72 °C for 1 min for extension. Finally, the PCR products were incubated at 72 °C for 10 min. Amplified PCR products were analyzed on 1% agarose gel (Fisher Scientifific, Loughborough, UK). Amplicons were sequenced by Shanghai Sangon Bioengineering using an ABI 3730 sequencer (Applied Biosystems®) with the same primers as used for PCR amplification.

**Table 2:** Primer sequences of the target genes

Gene	Primer Sequence(5′ → 3′)	Size (bp)	Reference
*qacEΔ1*	F: TAGCGAGGGCTTTACTAAGC	300	[12]
	R: ATTCAGAATGCCGAACACCG		
*qacA/B*	F: CTATGGCAATAGGAGATATGGTGT	416	[3]
	R: CCACTACAGATTCTTCAGCTACATG		
*cepA*	F: CAACTCCTTCGCCTATCCCG	1051	[12]
	R: TCAGGTCAGACCAAACGGCG		

### Statistical analysis

The MICs and MBCs were analysed by *Manne-Whitney* test. Differences in MIC and MBC distribution were compared by testing for equality of populations using the *Kruskal-Wallis* test. Presentation of resistance genes results was analysed by the unpaired *t*-test. Differences with a *P*-value of < 0.05 were considered to be statistically significant.

## Results

### Antimicrobial susceptibility profile

Among the 50 strains of carbapenem-resistant *Enterobacteriaceae* (CRE) with ertapenem MICs≥2 μg/ml, one strain of *K. pneumoniae* showed susceptible to imipenem with MIC = 1 μg/ml. Most of the agents exhibited very high resistance rates (Table 3).

**Table 3:** Antimicrobial susceptibility test results of the 50 strains of carbapenem-resistant *Enterobacteriaceae* (CRE)

Antibiotics	CRKP(*n* = 36)			CREC(*n* = 14)		
	Resistant	Intermediate	Susceptible	Resistant	Intermediate	Susceptible
ETP	100%	0	0	100%	0	0
IMP	94.4%	2.78%	2.78%	100%	0	0
FEP	100%	0	0	100%	0	0
CAZ	100%	0	0	100%	0	0
TZP	100%	0	0	100%	0	0
AMK	86.1%	0	13.9%	92.9%	0	7.14%
FOX	100%	0	0	100%	0	0
SAM	100%	0	0	100%	0	0
LEV	94.5%	0	5.55%	85.7%	0	14.3%
CIP	97.2%	0	2.78%	85.7%	0	14.3%
CTX	100%	0	0	100%	0	0
CRO	100%	0	0	100%	0	0

*ETP* ertapenem, *IMP* imipenem, *FEP* cefepime, *CAZ* ceftazidime, *TZP* piperacillin-tazobactam, *AMK* amikacin, *FOX* cefoxitin, *SAM* ampicillin-sulbactam, *LEV* levofloxacin, *CIP* ciprofloxacin, *CTX* cefotaxime, *CRO* ceftriaxone

### Genetic diversity

**Fig. 1:**
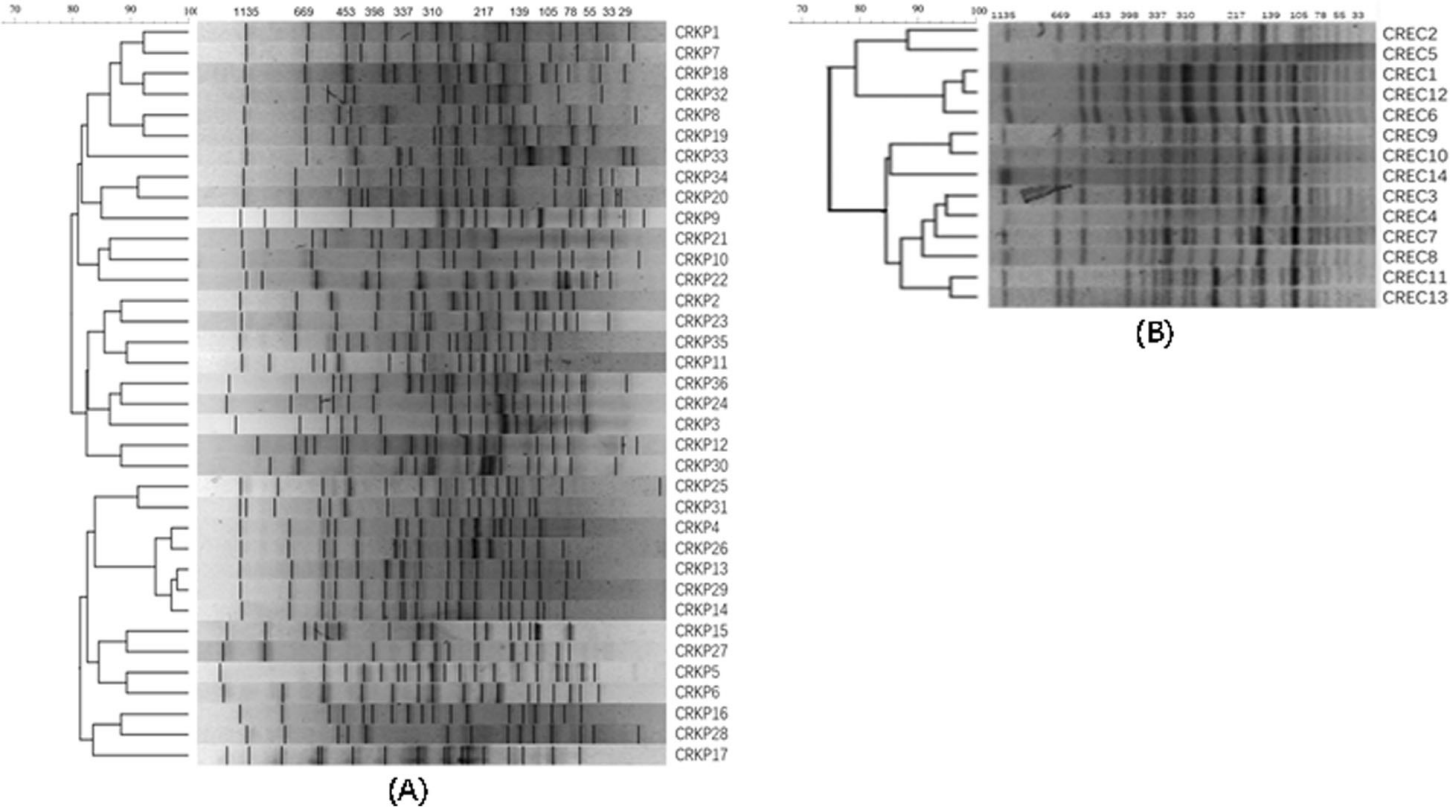
PFGE typing of 50 CRE strains. **a** Cluster analysis of 36 carbapenem-resistant *K. pneumoniae* (CRKP) strains; **b** Cluster analysis of 14 carbapenem-resistant *E. coli* (CREC) strains

In Fig. 1, it showed the PFGE typing from 50 CRE strains. The 36 CRKP were divided into 16 unique PFGE types, which suggests that the majority (16/36) of the isolates were considered distinct as they demonstrated < 85% similarity with any other isolate. The 14 CREC strains belonged to 5 different PFGE clusters, indicating that they were disseminated horizontally through the population and not just by the spread of a single strain.

### Sensitivity of the clinically isolated strains to each disinfectant

In general, compared with the reference strains, we observed higher MICs and MBCs of chlorhexidine and PVP-I (Table 4). Specially, the MICs of chlorhexidine against CRKP and CREC ranged from 8 to 512 mg/L(MIC_90_ = 32 mg/L), and 4 to 128 mg/L(MIC_90_ = 16 mg/L), respectively, which were generally higher than those observed for *K. pneumoniae* ATCC 700603 and *E. coli* ATCC 25922 reference strains (16 mg/L and 2 mg/L, respectively). There were significant differences in MIC and MBC distribution between the CRKP(CREC) strains and CSKP(CSEC) strains against chlorhexinine and PVP-I (*p < 0.05*). Moreover, against chlorhexidine or PVP-I, the MIC_90_ of 36 CRKP strains was higher than that of 50 CSKP strains (*p < 0.05*), suggesting a decreased sensitivity of carbapenem-resistant strains against the common used disinfectants.

**Table 4:** Sensitivity of the clinically isolated strains to each of the disinfectants

Disinfectants	Chlorhexidine			TCCA			PVP-I		
	MIC	MIC_90_ (mg/L)	MBC	MIC	MIC_90_ (mg/L)	MBC	MIC	MIC_90_ (mg/L)	MBC
Isolates	(mg/L) range		(mg/L) range	(mg/L) range		(mg/L) range	(mg/L) range		(mg/L) range
CRKP (*n* = 36)	8~512	32	8~512	64~128	128	64~256	8~128	32	8~128
CSKP (*n* = 50)	8~256	16	8~256	64~128	128	64~128	4~64	16	4~64
*ATCC* *700,603*	16		16	128		128	16		16
CREC (*n* = 14)	4~128	16	4~128	64~128	128	64~128	4~128	32	4~128
CSEC (*n* = 30)	2~128	8	2~128	64~128	128	64~128	4~64	32	4~64
*ATCC* *25,922*	2		2	128		128	32		32

### PCR detection of relevant drug-resistance genes *qacEΔ1*, *qacA/B* and *cepA*

**Fig. 2:**
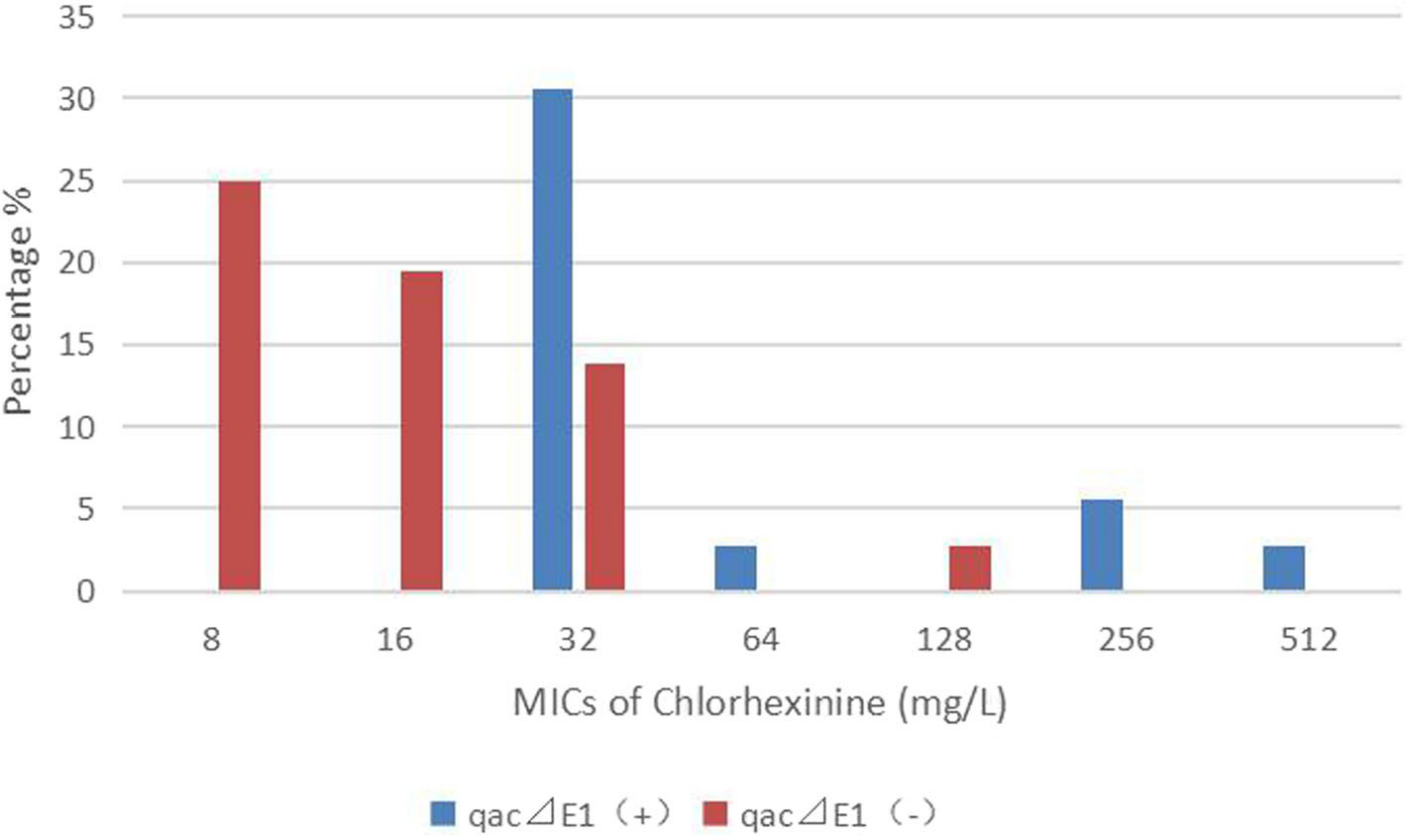
Distribution of the minimum inhibitory concentrations of chlorhexidine among 36 CRKP strains. Isolates carrying the *qacEΔ1* gene (blue bars) were significantly less susceptible to chlorhexidine than those without carrying *qacEΔ1* gene (red bars) [*p < 0.05*]

**Fig. 3:**
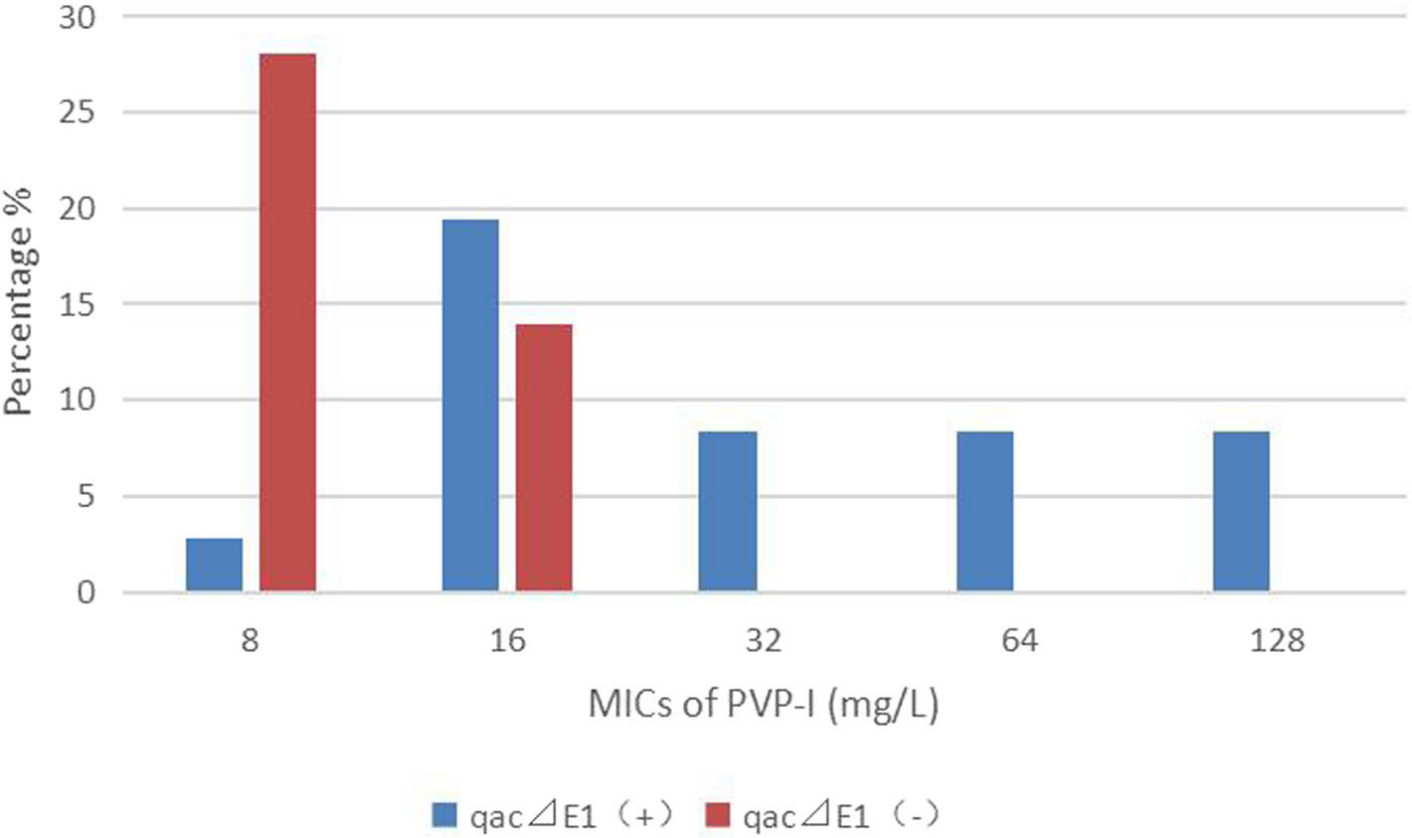
Distribution of the minimum inhibitory concentrations of PVP-I among 36 CRKP strains. Isolates carrying the *qacEΔ1* gene (blue bars) were significantly less susceptible to PVP-I than those without carrying *qacEΔ1* gene (red bars) [*p < 0.05*]

Among 36 CRKP strains, 41.7% (15/36) of them were positive for *qacEΔ1*, and 80.6% (29/36) for *cepA,* which was much higher than that in CSKP group (*p < 0.05*) (Table 5). Among 14 CREC strains, the *qacEΔ1* and *cepA* genes were more frequently amplified in 8 (57.1%) and 7 (50.0%) than CSEC group (*p < 0.05*), respectively. None of the tested CRE strains were positive for *qacA/B*. Specially, isolates carrying the *qacEΔ1* gene were significantly less susceptible to chlorhexidine and PVP-I than those without carrying *qacEΔ1* gene (*p < 0.05*) (Figs. 2 and 3).

**Table 5:** Detection of the disinfectant-resistance genes among CRE strains and non-CRE strains

	*qacEΔ1*		*qacA/B*		*cepA*	
CRKP	41.7% (15/36)	*p > 0.05*	0	–	80.6% (29/36)	*p < 0.05*
CSKP	36.0% (18/50)		0		58.0% (29/50)	
CREC	57.1% (8/14)	*p < 0.05*	0	*p > 0.05*	50.0%(7/14)	*p < 0.05*
CSEC	33.3% (10/30)		3.33% (1/30)		33.3% (10/30)

## Discussion

In this study, 50 CRE strains all exhibited resistance to ertapenem as well as the third and fourth generation of cephalosporins. In the clinic, there are limited options of antibiotic drugs available for treating CRE infections currently. Thus, the consequences of an outbreak may be serious, and effective strategies to fight against the presence of CRE in the hospital environment are essential to control the spread of this infection.

In the present study, we chose three clinically common used disinfectants, chlorhexidine acetate, trichloroisocyanuric acid and PVP-I, which are topical disinfectants with a broad spectrum of activity. They are widely used in hospitals in different applications such as hand hygiene, skin preparation before invasive operation and surface cleaning [13–15]. Significantly, it is very likely that, the same as the emergence antibiotic resistance, the drug resistant strains will also gradually grow resistant to the clinically common used disinfectants [3]. Indeed, there were significant differences in MIC and MBC distribution between the CRKP(CREC)strains and CSKP(CSEC)strains against chlorhexinine and PVP-I in this study, suggesting a decreased sensitivity of carbapenem-resistant strains against the common used disinfectants.

The presence of the *qacEΔ1*and *cepA* genes plays a potential role on increasing the level MICs against disinfectants [6]. It is reported that the genes, *qacEΔ1* and *cepA*, have a close relationship with decreasing antiseptic susceptibility in *Enterobacteriaceae* strains [12]. Usually, the *qacEΔ1* gene was located upstream of the *sul1* sulfonamide resistance gene and downstream of the aminoglycoside adenyltransferase gene (*aadA1*) directly, which was flanked by the dihydrofolate reductase gene *dhfrA1*. The *qacEΔ1* gene seems to be part of a small resistance island indicating that this gene is related and migrates with antibiotic resistance genes. The close relationship of *qac* genes to antibiotic resistance genes has been proved previously in resistance islands. The widespread carriage of *qac* genes in *K. pneumoniae* [6] and their linkage to antibiotic resistance genes suggests that excessively use of antiseptics could select antibiotic-resistant strains.

Our study demonstrated that over 40% of the CRE strains carried the two genes *qacEΔ1* and *cepA*. Specifically, the *cepA* gene was much more prevalent than the *qacEΔ1*; it reached over 80% among CRKP strains. Compared to the CSKP strains, the presence of the *qacEΔ1* and *cepA* genes was significantly higher among the CRKP strains, suggesting that CRKP strains harbouring drug-resistance genes might have potentially higher tolerance to growth inhibition or killing by disinfectants than those susceptible strains.

Chlorhexidine is a cationic biguanide antiseptic. In present study, the MIC values of CRKP and CREC against chlorhexidine were found to be 8 to 512 mg/L, and 4 to 128 mg/L, respectively. Previous reports have described reduced susceptibility to chlorhexidine among *K. pneumoniae* strains; but the most frequently reported MIC, using the agar dilution method, was 32 μg/mL [7, 13], which was consistent with our result. In the study of Naparstek et al. [16], it was reported that 90 % of ST258 *K. pneumoniae* isolates had an MIC of chlorhexidine of > 128 mg/L. Against *Escherichia coli,* we found that the susceptibility of chlorhexidine has decreased compared with previous studies [17, 18], since the MIC distribution observed in this study was 4 to 128 mg/L (CREC), and 2 to 128 mg/L (CSEC). Most notably, the CRKP strains carrying *qacEΔ1*gene showed less susceptible against chlorhexidine, suggesting there was a linkage between *qacEΔ1* gene and antibiotic resistance genes. It is believed that widespread use of biocides, particularly as antiseptics, could select antibiotic-resistant strains [8, 19]. However, the role of *cepA* gene on chlorhexidine resistance is ambiguous. Fang et al. [7] found that the *cepA* efflux pump is associated with reduced susceptibility to chlorhexidine. Abdulmonem & Sebastian revealed that as the MIC of chlorhexidine increased, so did the expression of *cepA* [12]. Yet, Naparstek et al. [16] did not find a correlation between chlorhexidine susceptibility and *cepA* gene expression.

TCCA is a chlorinated derivative of isocyanurate, with high content of chlorine. It has a strong and long-lasting sterilization effect. In China, it is widely used in medical health care systems, especially for disinfection of medical devices, equipment, and environment. To our knowledge, this present study is the first study reported the susceptibility of CRE against TCCA in China. We determined that the MIC values of 50 CRE strains against TCCA was 64 to 128 mg/L. In this study, there was no close relationship reported between the resistance genes (*qacEΔ1*, *qacA/B* and *cepA*) and the TCCA susceptibility. Certainly, there is no doubt that it is of significance to keep monitoring the tendency of the reduced susceptibility against disinfectants.

Povidone-iodine (PVP-I), also known as iodopovidone, is an antiseptic used for skin disinfection before and after surgery. At present, there are few studies on the disinfection effect of iodine on drug-resistant bacteria, especially on CRE [20, 21]. Our study will help fill this gap. This study showed the MIC_90_ of 0.1% PVP-I against the 36 strains of CRKP and 14 strains of CREC was 32 mg/L, which was consistent with the result of Guo et al. [3] Moreover, among the 36 CRKP isolates, those carrying *qacEΔ1* gene were significantly less susceptible to 0.1% PVP-I than those without *qacEΔ1* gene, showing that the *qacEΔ1* gene might play a certian role on the mechanism of resistance to iodophor, which needs further investigation.

However, there are certain limitations in the present study. First of all, the sample size was small. More CRE strains are needed for further solid statistical analysis. Second, we provided the necessity for determining the susceptibility of CRE strains against common disinfectants, but the efficacy of the tested disinfectants in eliminating each of these clinically isolated CRE strains from the real-world hospital settings was not evaluated (i.e. on surfaces or medical equipment), which would be further investigated.

## Conclusion

CRE strains collected from patients in our hospital exhibit various degree of resistance to the commonly used disinfectants. CRE strains were highly tolerant to disinfectants, with a higher distribution of disinfectant-resistance genes. Incorrect and excessive use of disinfectants has imposed selective pressure on strains, resulting in the high level of resistance to disinfectants and the wide distribution of resistant genes [22]. This study suggested that, priority should be given to monitoring the disinfectants resistance rate of CRE strains in the hospital environment, to ensure that appropriate and effective disinfection measures are taken in the hospital environment to prevent the spread of these life-threatening resistant strains.

## Data Availability

All data generated or analyzed during this study are included in this published article. The datasets used and/or analysed during the current study are available from the corresponding author on reasonable request.

## Abbreviations

CRE: Carbapenem-resistant *Enterobacteriaceae*
CREC: Carbapenem resistant *Escherichia coli*
CRKP: Carbapenem resistant *Klebsiella pneumoniae*
MBC: Minimum bactericidal concentration
MIC: Minimum inhibitory concentration
PCR: Polymerase chain reaction
